# Rheumatoid Factor Positivity Is Associated with Increased Joint Destruction and Upregulation of *Matrix Metalloproteinase 9* and *Cathepsin K* Gene Expression in the Peripheral Blood in Rheumatoid Arthritic Patients Treated with Methotrexate

**DOI:** 10.1155/2013/457876

**Published:** 2013-11-14

**Authors:** Elena V. Tchetina, Natalia V. Demidova, Dmitry E. Karateev, Eugeny L. Nasonov

**Affiliations:** ^1^Clinical Immunology Department, Nasonova Research Institute of Rheumatology, Russian Academy of Medical Sciences, Kashirskoye Shosse 34A, Moscow 115522, Russia; ^2^Early Rheumatoid Arthritis Department, Nasonova Research Institute of Rheumatology, Russian Academy of Medical Sciences, Moscow 115522, Russia; ^3^Vascular Pathology Department, Nasonova Research Institute of Rheumatology, Russian Academy of Medical Sciences, Moscow 115522, Russia

## Abstract

We evaluated changes in gene expression of *mTOR*, *p21*, *caspase-3*, *ULK1*, *TNF**α**, matrix metalloproteinase* (*MMP)-9*, and *cathepsin K* in the whole blood of rheumatoid arthritic (RA) patients treated with methotrexate (MTX) in relation to their rheumatoid factor status, clinical, immunological, and radiological parameters, and therapeutic response after a 24-month follow-up. The study group consisted of 35 control subjects and 33 RA patients without previous history of MTX treatment. Gene expression was measured using real-time RT-PCR. Decreased disease activity in patients at the end of the study was associated with significant downregulation of *TNF**α*** expression. Downregulation of *mTOR* was observed in seronegative patients, while no significant changes in the expression of *p21*, *ULK1*, or *caspase-3* were noted in any RA patients at the end of the study. The increase in erosion numbers observed in the seropositive patients at the end of the follow-up was accompanied by upregulation of *MMP-9* and *cathepsin K*, while seronegative patients demonstrated an absence of significant changes in *MMP-9* and *cathepsin K* expression and no increase in the erosion score. Our results suggest that increased expression of *MMP-9* and *cathepsin K *genes in the peripheral blood might indicate higher bone tissue destruction activity in RA patients treated with methotrexate. The clinical study registration number is 0120.0810610.

## 1. Introduction

Rheumatoid arthritis (RA) is a chronic inflammatory disease characterized by synovial hyperplasia, mononuclear cell infiltration, bone erosion, and joint destruction. Early diagnosis and immediate aggressive treatment are required for the amelioration of progressive joint damage and patient disability [1, 2].

Methotrexate (MTX) is the most conventional disease-modifying antirheumatic drug (DMARD) for RA, with the best efficacy and the fewest adverse effects [3, 4]. However, only approximately 30% of patients respond to MTX treatment [5, 6]. The identification of patients who are less responsive to MTX could avoid delays in adjusting their treatment and prevent future irreversible joint damage [7].

Rheumatoid factor (RF) is a part of the 2010 American College of Rheumatology (ACR) classification criteria for RA [8]. RF is an autoantibody directed against the Fc portion of IgG and is associated with disease persistence and progressive joint destruction [9–11]. However, the data related to RF status in treatment response to MTX is inconsistent, as some studies reported no association between RF positivity and treatment efficacy [12–21], while others indicated that seropositive patients exhibited worse responses to MTX therapy in early rheumatoid arthritis [9, 22, 23].

Variations in disease manifestations assessed by clinical and laboratory tests produce a specific disease phenotype, which results in changes in gene expression in various affected tissues and immune effector cells [24]. Therefore, differentially expressed genes may serve as biomarkers for disease status and predictors of the response to therapy [25–28]. As the peripheral immune system is activated in RA patients [29], gene expression changes in the peripheral blood mononuclear cells (PBMCs) could provide informative biomarkers. Several studies involving DNA microarray technology have revealed differences in the expression of specific gene clusters observed in the PBMCs of early RA patients and in patients with established progressive disease versus normal subjects [30]. Higher expression of type I interferon-regulated genes was also observed in the peripheral blood cells of RA patients compared with healthy controls [31]. In addition, twin studies have shown that similar genes are highly overexpressed in both blood and synovial fluid of RA patients versus controls [32].

Of particular importance are ubiquitously expressed human genes that are required for the regulation of basic cellular processes [33]. Previous studies have revealed differential gene expression associated with apoptosis in the PBMCs of RA patients [25, 34]. In addition, low apoptotic activity has been reported in the synovial fluid leucocytes and synoviocytes of RA patients [35–37].

Mammalian target of rapamycin (*mTOR*) is considered a key regulator of cell growth and proliferation [38]. It has been shown recently that *mTOR* inhibition downregulated mitogen-induced T- and B-lymphocyte proliferation and IL-1 and *TNF*α** production in vitro [39, 40]. Moreover, animal studies have shown that *mTOR* downregulation alleviated paw swelling in antigen-induced arthritis [41].

Autophagy occurs upon arrest of proliferation and is associated with production of cyclin-dependent kinases such as *p21* [42]. As autophagy can also be induced by proinflammatory cytokines and autoantibodies, it could be an important factor in RA pathogenesis [43]. Indeed, it has been shown that autophagy induction in RA synovial fibroblasts promoted their survival [44].

Several studies have presented evidence of upregulated proteolytic activity in the PBMCs of RA patients versus healthy subjects [32], which might result from joint destruction in RA. Articular cartilage and bone degradation are associated with the upregulation of *matrix metalloproteinases* (*MMPs*) and osteolytic enzymes, such as *MMP-9* and *cathepsin K*, respectively [45–47], in the serum and synovial fluid of RA patients [48, 49]. Moreover, serum concentrations of *cathepsin K* significantly correlated with radiological joint destruction in RA patients [50]. *MMP-9* expression is activated by proinflammatory cytokines including *TNF*α** [51] and has been shown to be both decreased [52, 53] and increased [54] in response to anti-TNF therapy.

Here, we evaluated changes in the expression of genes responsible for cell proliferation and growth (*mTOR*), regulation of cell cycle progression (*p21*), apoptosis (*caspase-3*), and autophagy (*ULK1*), as well as the proinflammatory cytokine *TNF*α** and genes associated with bone and articular cartilage turnover (*MMP-9* and *cathepsin K*) in the whole blood of rheumatoid arthritic patients treated with MTX in relation to their RF status, clinical, immunological, and radiological parameters, and their therapeutic response at a 24-month follow-up. Our results suggest that the higher radiographic joint destruction associated with RF positivity is accompanied by the upregulation of *MMP-9* and *cathepsin K* gene expression in the PBMCs of RA patients treated with methotrexate.

## 2. Materials and Methods

### 2.1. Ethics

Our clinical study was in compliance with the Helsinki Declaration. The study protocol was approved by the Local Committee on the Ethics of Human Research, and informed consent was obtained from all subjects.

### 2.2. Patients

Inclusion criteria of the control subjects were as follows. The control group consisted of 35 subjects, 7 men and 28 women, (average age 46.4 ± 13.2 years; range 19–69 years) with no current chronic or acute infection and no family history of autoimmune diseases.

Inclusion criteria of the RA patients were as follow. The RA patient group consisted of 33 consecutive, unrelated rheumatoid arthritic patients, 5 men and 28 women (average age 47.2 ± 14.2 years; range 18–68 years), who visited the clinic of the Institute of Rheumatology, Russian Academy of Medical Sciences, between January and December 2008. Inclusion criteria involved a diagnosis of RA, as defined by the American College of Rheumatology (ACR) 1987 [55], age ≥ 18 years, and symptom duration of <2 years without previous history of MTX treatment. Exclusion criteria were previous treatment with DMARDS and/or systemic corticosteroids and DMARD intolerance.

All patients included in this study started treatment with oral MTX at a dosage of 10 mg per week; after two weeks, the dosage was increased to 15 mg. Out of 33 patients, 11 were given MTX in combination with methylprednisolone, 8 mg daily. Each patient was followed up by the same investigator at six months, one year, and two years after inclusion. Remission was defined according to ACR criteria for clinical remission by the disease activity score based on the simplified 28-joint score (DAS28) [56, 57].

### 2.3. Demographic, Clinical, and Immunologic Assessment

The evaluation data were collected at baseline and at 24 months. These data included age, gender, disease duration, Steinbrocker's radiographic stage [58], duration of morning stiffness (min), and the disease activity score (DAS) using a modified index involving 28 joints [56, 57]. Concentrations of serum C-reactive protein (cutoff value, 5 mg/L) and IgM class rheumatoid factor (RF) (a standard cutoff value of 15 mU/L was used) were measured by nephelometry using a BN-100 analyzer (Dade Bering, Germany). Anticitrullinated protein autoantibodies (ACPA) were detected by ELISA according to the manufacturer's recommendations (the cutoff level was set at 5 U/mL for antibody positivity) (Axis Shield Diagnostics Limited, UK).

### 2.4. Radiographic Assessment

Radiographs of hands and feet were obtained at months 0 and 24. The radiographs were evaluated blind and in chronological order by two independent observers and scored using Sharp's method as modified by van der Heijde et al. [59]. For each patient, an erosion and joint space narrowing score was registered for hands and feet, and the mean of the scores from two observers was used to determine the final radiographic scores for erosions and joint space narrowing.

### 2.5. Total RNA Isolation and Reverse Transcriptase (RT) Reaction

For detection of gene expression total RNA was isolated from 100 *μ*L of whole blood immediately after withdrawal using Ribo-zol-A kit (InterLabService, Moscow, Russia) in accordance with the manufacturer's recommendations. Total RNA had an A_260/290_ > 1.9. The RT reaction was performed using a Reverta kit containing M-MLV reverse transcriptase, random hexanucleotide primers, and total RNA according to the manufacturer's recommendations (InterLabService, Moscow, Russia).

### 2.6. Real-Time Quantitative PCR

The following premade primers and probes were used for the TaqMan assay (Applied Biosystems, Foster City, CA, USA): *mTOR* (Hs00234522_m1), *Unc-51-like kinase 1* (*ULK1*) (Hs00177504_m1), *p21WAF1/Cip1* (*p21*) (Hs00355782_m1), *caspase 3* (Hs00263337_m1), *TNF*α** (Hs00174128_m1), *MMP-9* (Hs00234579_m1), and *cathepsin K* (Hs00166165_m1). *β-Actin* was used as an endogenous control.

The quantification of gene expression was conducted using a 7300 Real-Time PCR System (Applied Biosystems, Foster City, CA, USA) as described previously [60]. Briefly, 1 *μ*L of RT product was subjected to real-time PCR in a 15 *μ*L total reaction mixture containing 7.5 *μ*L of TaqMan Universal PCR Master Mix (Applied Biosystems), 900 nM sense and antisense primers, 50 nM probe, and template cDNA. After a single step of 50°C for 2 min and an initial activation at 95°C for 10 min, the reaction mixtures were subjected to 40 amplification cycles (15 s at 95°C for denaturation and 1 min of annealing and extension at 60°C).

Relative mRNA expression was determined using the delta-delta C_T_ method, as detailed by the manufacturer guidelines (Applied Biosystems) [61]. The delta C_T_ value was calculated by subtracting the C_T_ value for the housekeeping *β-actin* gene from the C_T_ value for each sample. A delta-delta C_T_ value was then calculated by subtracting the delta C_T_ value of the control (each healthy patient) from the delta C_T_ value of each RA patient. Each PCR was performed in duplicate. Three “no template” controls were consistently negative for each reaction.

### 2.7. Statistical Analysis

The variables did not have a Gaussian distribution; therefore, descriptive values were expressed as medians and interquartile ranges. The statistical comparison between the independent patient groups was performed using Mann-Whitney *U* test and Spearman's rank correlations. For the statistical comparison between the RA patient groups before and after treatment the Wilcoxon matched pairs test was applied. To compare percentages, a one-tailed *Z*-test for percentages was applied. The Statistica 6 Software (StatSoft, Tulsa, OK, USA) was used for all statistical analyses. *P*  values ≤ 0.05 were considered significant.

## 3. Results

### 3.1. Whole Blood Gene Expression in Rheumatoid Arthritic Patients at Baseline and at 24 Months

All of examined genes, the regulator of cell growth and proliferation *mTOR,* the autophagy marker *ULK1*, the cyclin-dependent kinase inhibitor *p21*, the apoptosis indicator *caspase-3*, the proinflammatory cytokine *TNF*α*,* and the proteases *MMP-9* and *cathepsin K,* were significantly upregulated at baseline in a sample of RA patients (*n* = 33) compared with healthy subjects (data not shown).

An analysis of bivariate correlations using Spearman's correlation coefficient for the expression of the examined genes at baseline showed positive correlations (*P* < 0.05) with each other in the RA patients examined (*n* = 33) (Table 1). However, no correlation was observed between the expression of *mTOR* and *TNF*α** and that of *MMP-9*. A positive correlation was also noted between *ULK1* and *MMP-9* gene expression and serum C-reactive protein levels. In contrast, expression of the *p21, caspase-3*, and *TNF*α** genes negatively correlated with serum RF amounts. As RF concentration correlated with the expression of the examined genes, the RA patients were divided into seronegative and seropositive subsets for further analyses.

**Figure 1:**
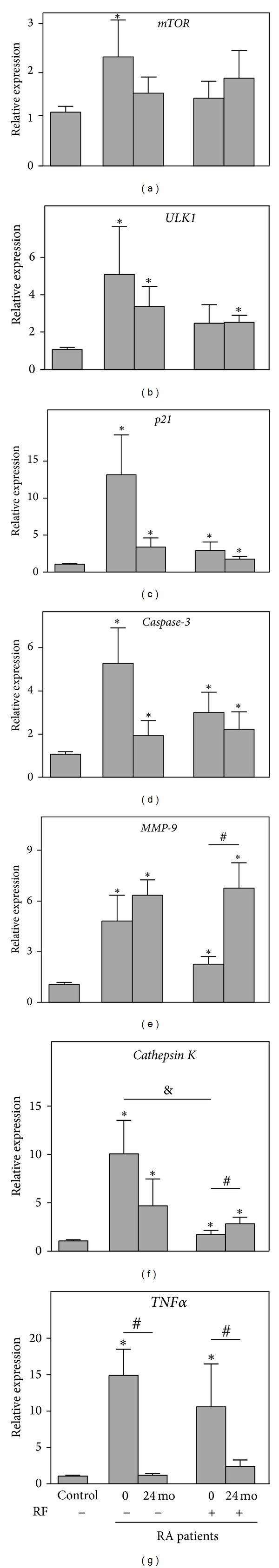
Relative expression of the genes *mTOR* (a), *ULK1* (b), *p21* (c), *caspase-3* (d), *MMP-9* (e), and *cathepsin K* (f), and *TNF*α** (g) with reference to *β*-*actin* determined by real-time PCR analyses in the whole blood of seronegative (RF−) (*n* = 12) and seropositive (RF+) (*n* = 21) rheumatoid arthritic patients compared with healthy controls (Control) (*n* = 35) at baseline (0) and after 24 months of follow-up (24 mo). Control bar is shown as 1.0 as required for relative quantification with the real-time PCR protocol. Asterisks indicate significant differences from the control in pairwise comparisons (Mann-Whitney *U* test). Number sign (#) shows significant difference from the baseline value (Wilcoxon matched pairs test). & sign indicates significant difference between seronegative and seropositive RA patients (Mann-Whitney *U* test).

Examination of gene expression in the blood of 12 seronegative RA patients revealed that all of examined genes were significantly upregulated at baseline compared to healthy controls (Figure 1). At the end of the study, downregulation was observed only for the *TNF*α** (*P* = 0.03 versus baseline) and *mTOR* genes, the expression of which became similar to that in the control subjects. Some decrease in the expression of *ULK1, p21*, *caspase-3*, and *cathepsin K* was also observed; however, these differences were not statistically significant, and the expression of these four genes exceeded that observed in the healthy controls.

Assessment of gene expression in the blood of 21 seropositive RA patients showed that their *mTOR* and *ULK1* levels were similar to those in healthy subjects at baseline, while the other examined genes, *p21*, *caspase-3*, *TNF*α*, MMP-9,* and *cathepsin K,* were significantly upregulated (Figure 1). At the end of the study, no significant changes were observed in the expression of *ULK1, p21,* and *caspase-3*, as these genes remained upregulated compared with the controls. In contrast, the expression of *MMP-9* and *cathepsin K* was significantly upregulated versus that at baseline (*P* = 0.02 and *P* = 0.05, resp.) and compared with the controls, while *TNF*α** gene expression was significantly decreased compared with baseline (*P* = 0.05).

Direct comparison of gene expression between RF-positive and RF-negative RA patients showed that, at baseline, the seronegative subjects exhibited significantly higher *cathepsin K* gene expression compared with seropositive RA patients (*P* = 0.02), while the expression of the remaining genes was not significantly different (Figure 1(f)). Additionally, no significant differences in gene expression were observed between RF-positive and RF-negative patients at the end of the follow-up.

### 3.2. Clinical, Immunological, and Radiological Parameters at Baseline and 24 Months and Therapeutic Response in Rheumatoid Arthritic Patients

The mean age at diagnosis of the enrolled seronegative (*n* = 12) rheumatoid arthritic patients was 49.0 ± 15.5 (range 18–66 years). The subset included one man and 11 women. The mean disease duration at inclusion was 4.3 ± 4.2 months. Of 12 examined patients, 11 had early RA, with disease duration from 1 to 5 months; the disease duration of one patient was 17 months. All of the patients had Steinbrocker's radiographic stage II, both at baseline and after 24 months of follow-up. All of the patients were treated with MTX, while 5 out of 12 received a combination of MTX and methylprednisolone. Only 3 out of 12 patients were ACPA-positive both at baseline and after 24 months. Most patients (8 out of 12) presented high disease activity (DAS28 > 5.1) at study entry (Table 2). The DAS28 index decreased significantly after 24 months of follow-up (*P* = 0.001). At the end of the study, the majority of patients had moderate disease activity (3.2 < DAS28 < 5.1), while four patients (33%) fulfilled the remission criteria (DAS28 < 2.6). These findings were associated with significant decreases in morning stiffness and in the number of swollen and tender joints. Only one patient out of 12 exhibited erosions both at the beginning and at the end of the study, while the joint space narrowing score increased over the course of the follow-up period (*P* = 0.003).

The mean age at diagnosis of the seropositive (*n* = 21) rheumatoid arthritic patients was 46.2 ± 13.7 (range 18–68 years). This subset included 4 men and 17 women. The mean disease duration at inclusion was 9.2 ± 6.5 months; a total of 18 patients had early RA, with a disease duration from one to 12 months, while four patients had established RA with a disease duration of 18–23 months. All of the patients had Steinbrocker's radiographic stage II at baseline. After 24 months of follow-up, 18 patients maintained stage II, while three patients developed radiographic stage III. All of the patients were treated with MTX, while 6 out of 21 were treated with a combination of MTX and methylprednisolone. Only 3 out of 21 patients were ACPA-negative both at baseline and after 24 months of follow-up. Most patients (15 out of 21) presented high disease activity (DAS28 > 5.1) at baseline (Table 3). The DAS28 index decreased significantly after 2 years (*P* = 0.0002). At the end of the study, the majority of patients had moderate disease activity (3.2 < DAS28 < 5.1) while five patients (24%) fulfilled the remission criteria (DAS28 < 2.6). These findings were associated with significant decreases in morning stiffness and in the numbers of swollen and tender joints. Five patients out of 21 exhibited erosions at the beginning of the study; by the end of the study, 10 patients out of 21 had erosions, and the erosion score significantly increased (*P* = 0.003) after two years of follow-up. The joint space narrowing score also increased significantly over the course of the follow-up period (*P* = 0.001).

Direct comparison of the clinical, immunological, and radiological parameters between the groups showed that, at baseline, seropositive patients exhibited significantly higher RF (*P* < 0.001) and ACPA (*P* = 0.01) values compared with seronegative RA subjects. After 24 months the seropositive RA patients also showed significantly elevated RF (*P* < 0.001) and ACPA (*P* = 0.006) versus seronegative subjects. In addition, at the end of the study, seropositive patients exhibited higher joint space narrowing (*P* = 0.006) values compared with seronegative RA patients, and there was a higher number of seropositive patients than seronegative patients with bone erosions (*P* = 0.02). However, no significant differences in the manifestation of other examined parameters were noted between the analyzed groups of RA patients both at baseline and at the end of the follow-up period (Tables 2 and 3).

## 4. Discussion

Many aspects of a disease phenotype are produced by pathophysiological processes driven by genes and their products [62]. Therefore, comparison of gene expression signatures between RA patients and healthy subjects may reveal important insights into mechanistic differences and unravel the fundamental nature of the disease. Moreover, this approach might also be useful in the evaluation of the response to RA treatment, which is supposed to restore normal cellular metabolism and should arguably aim to restore gene expression to levels comparable to healthy controls.

As it has been noted that more homogenous patient groups produce more consistent results [63], we analyzed the value of RF status on the outcome of MTX therapy in a sample of RA patients during a 24-month follow-up in relation to changes in the expression of genes involved in basic cellular processes and joint function, as measured in the peripheral blood. We found that, in the majority of the examined patients, the disease activity in the subsets of seropositive and seronegative RA patients significantly decreased from high levels at baseline to moderate levels at the end of the follow-up. This decrease was accompanied by a significant decrease in the morning stiffness and the number of swollen and tender joints in both subsets and a significant downregulation of *TNF*α** gene expression in the blood compared with baseline levels. Moreover, *TNF*α** gene expression became equal to that of control subjects. These results support previous observations that MTX treatment decreases *TNF*α** production in T cells from RA patients [64, 65].

RF status did not affect the remission frequency (*P* = 0.29) in the examined RA patients; in both subsets, the remission criteria were fulfilled by 24% of the examined seropositive patients and by 33% of the seronegative RA patients. The inability of RF positivity to predict the outcome based on clinical parameters has also been observed previously [12–15, 66, 67].

The significantly increased erosion and joint space narrowing scores observed at the end of the follow-up period in the seropositive RA patients support previous observations that many RA patients exhibit radiographic progression, even though clinically they are in a state of low disease activity [68, 69]. Worsening of radiological parameters in these patients at the end of the study was associated with significant upregulation of *MMP-9* and *cathepsin K* gene expression in the peripheral blood compared with baseline values. In contrast, the less severe joint destruction noted in the seronegative RA patients was accompanied by fewer alterations in *MMP-9* and *cathepsin K *gene expression in the blood at the end of the follow-up. Therefore, upregulation of *MMP-9* and *cathepsin K* gene expression might serve as blood-based biomarker of increased joint destruction activity in RA patients treated with MTX.

The difference in the observed response to MTX treatment might be partially caused by ACPA positivity in the majority of the examined seropositive RA patients compared with seronegative subjects. Some studies have reported previously that ACPA positivity was related to resistance to DMARDs and was inversely associated with remission at 24 months [70, 71].

The decrease in the disease activity at the end of the follow-up was accompanied by downregulation of *mTOR* gene expression in seronegative RA patients to the level observed in healthy controls. This outcome is important in MTX therapy, as *mTOR* upregulation has been shown to be associated with interleukin (IL)-1 [72], *TNF*α** [40] production, synovial fibroblast proliferation [73], and osteoclast formation [74].

However, expression of the other examined genes (namely, *p21, caspase-3*, and *ULK1* which are also required for maintenance of basic cellular processes) did not show significant changes in RA patients over the course of treatment, remaining significantly upregulated compared with healthy controls at the end of the study. This result might indicate that MTX treatment does not ameliorate basic cellular functions associated with apoptosis, autophagy, and cell cycle control, which are disturbed in RA.

## 5. Conclusions

We have shown that a significant reduction in the disease activity of the examined RA patients treated with methotrexate during a 24-month follow-up was associated with the significant downregulation of *TNF*α** gene expression in the blood compared with baseline, which became equal to that in healthy subjects. Nevertheless, rheumatoid factor-positive RA patients exhibited significantly increased joint destruction accompanied by significant upregulation of *MMP-9 *and *cathepsin K* gene expression in the peripheral blood compared with baseline levels. These analyses may be of value in better characterizing disease activity and joint degeneration in rheumatoid arthritic patients.

## Figures and Tables

**Table 1:** Correlation coefficients (Spearman's) and their significance (*P*) are shown for the expression of the examined genes in relation to each other and the disease markers in a sample of RA patients (*n* = 33).

	*mTOR*	*ULK1*	*p21*	*Caspase-3*	*TNF*α**	*MMP-9*
*mTOR*		0.399 *P* = 0.01	0.702 *P* < 0.001	0.765 *P* < 0.001	0.581 *P* < 0.001	
*ULK1*			0.531 *P* = 0.001	0.539 *P* < 0.001	0.383 *P* = 0.02	
*p21*				0.915 *P* < 0.001	0.770 *P* < 0.001	
*Caspase-3*					0.632 *P* < 0.001	
*MMP-9*		0.661 *P* < 0.001	0.367 *P* = 0.03	0.389 *P* = 0.02		
*Cathepsin K*	0.634 *P* < 0.001	0.628 *P* < 0.001	0.708 *P* < 0.001	0.708 *P* < 0.001	0.688 *P* < 0.001	0.499 *P* = 0.002
Rheumatoid factor			−0.384 *P* = 0.02	−0.430 *P* = 0.009	−0.348 *P* = 0.04	
C-reactive protein		0.379 *P* = 0.02				0.303 *P* = 0.07

**Table 2:** Clinical, immunological, and radiological parameters and therapeutic response in seronegative rheumatoid arthritic patients.

	Baseline *n* = 12	24 months *n* = 12	*P* (Wilcoxon matched pairs *t*-test)
IgM RF, mU/mL	9.5 [9.5; 9.5]	9.5 [9.5; 9.5]	1.00
ACPA, U/mL	0.35 [0.15; 50.3]	1.0 [0.5; 44]	0.19
C-reactive protein, mg/L	12.51 [6.4; 30]	4.68 [1.5; 12.1]	0.001
DAS28	5.37 [4.5; 5.8]	3.29 [1.8; 3.5]	0.001
DAS28 < 2.6	0	4 (33%)	—
2.6 < DAS28 < 3.2	1 (8%)	1 (8%)	—
3.2 < DAS28 < 5.1	3 (25%)	7 (58%)	0.57
DAS28 > 5.1	8 (67%)	0	0.01
Morning stiffness, min	150 [75; 210]	10 [0; 30]	0.002
Swollen joints	8 [6; 10.5]	1 [0; 2]	0.001
Tender joints	8.5 [6.5; 11.5]	2 [0; 3]	0.001
Number of patients with erosions, %	8.3 (1/12)	8.3 (1/12)	—
Joint space narrowing score	8 [5.5; 11]	13 [8.5–17]	0.003

**Table 3:** Clinical, immunological, and radiological parameters and therapeutic response in seropositive rheumatoid arthritic patients.

	Baseline *n* = 21	24 months *n* = 21	*P* (Wilcoxon matched pairs *t*-test)
IgM RF, mU/mL	83.1 [58.5; 304.5]	76.7 [26.9; 250.1]	0.24
ACPA, U/mL	100 [20.3; 100]	100 [68.7; 100]	0.03
C-reactive protein, mg/L	13.8 [3.7; 20.8]	6.2 [4.4; 11.4]	0.03
DAS28	5.56 [4.7; 6.4]	3.5 [2.4; 4.1]	0.0002
DAS28 < 2.6	0	5 (24%)	—
2.6 < DAS28 < 3.2	1 (5%)	2 (9%)	0.30
3.2 < DAS28 < 5.1	5 (24%)	11 (52%)	0.34
DAS28 > 5.1	15 (72%)	3 (14%)	0.002
Morning stiffness, min	60 [30; 180]	16.5 [0; 27]	0.002
Swollen joints	8 [5.5; 13]	2 [0; 6]	0.001
Tender joints	9 [3; 18.5]	2.5 [0; 8]	0.006
Number of patients with erosions, %	23.8% (5/21)	47.6% (10/21)	0.05
Erosion score	0 [0; 0.5]	1.5 [0; 5]	0.003
Joint space narrowing score	13 [7; 24]	23 [15.5; 31.5]	0.001
